# Supplementary figures and images for: The Vibrio cholerae Quorum-Sensing Protein VqmA Integrates Cell Density, Environmental, and Host-Derived Cues into the Control of Virulence
Source: mBio. 2020 Jul 28;11(4):e01572-20. doi: 10.1128/mBio.01572-20 (PMC7387800; doi:10.1128/mBio.01572-20)

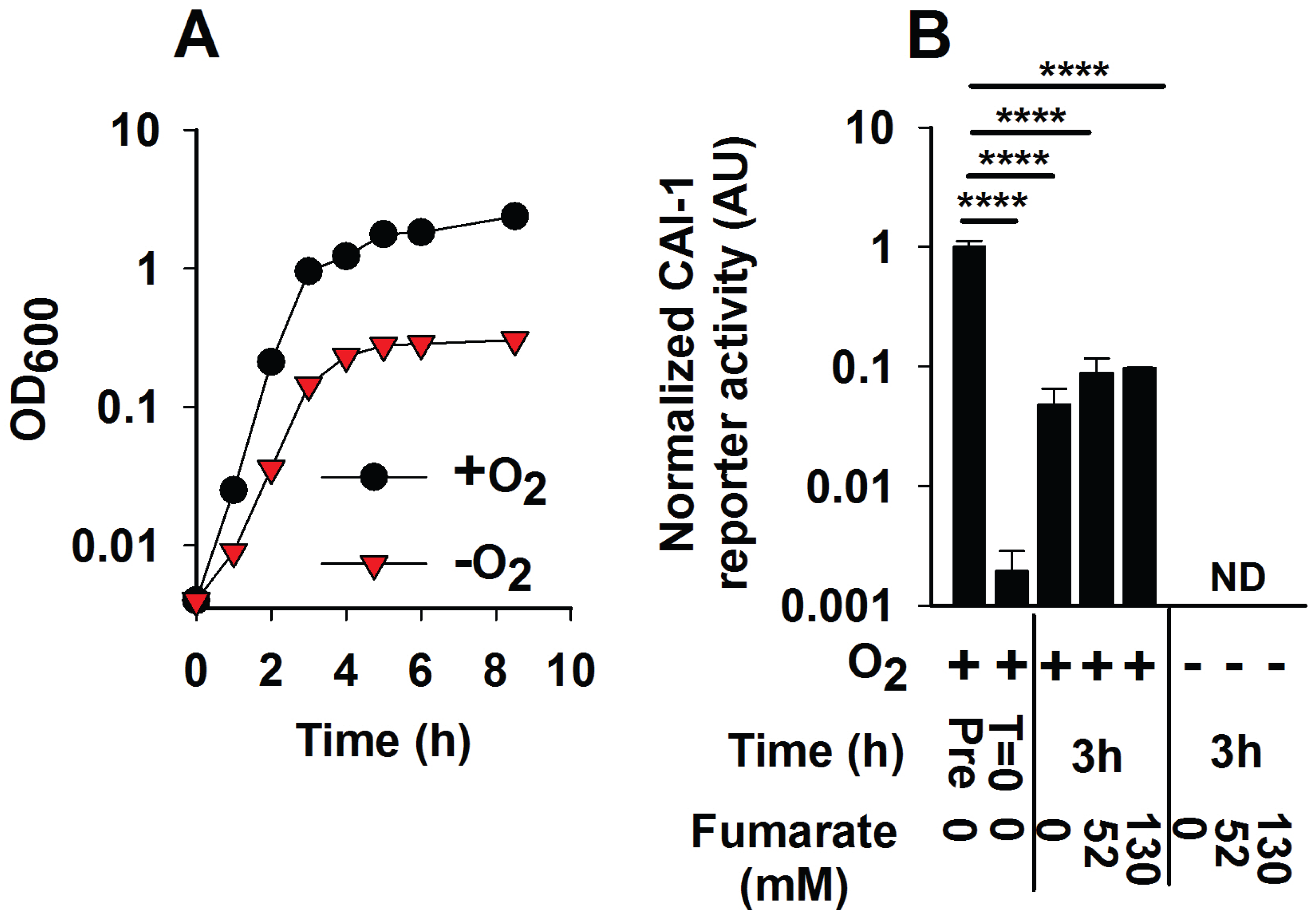

Supplement: FIG S1 [file mBio.01572-20-sf001.tif]

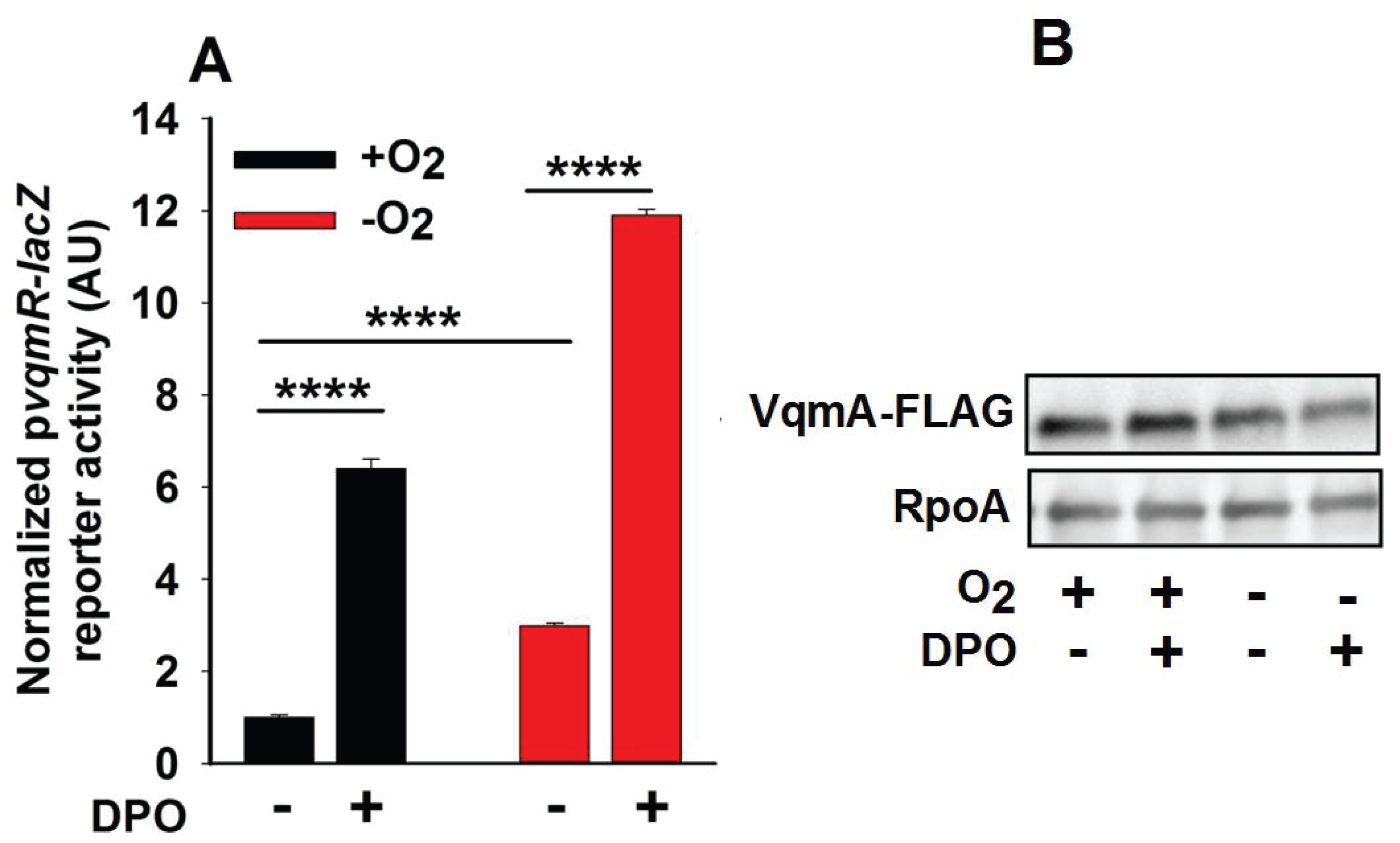

Supplement: FIG S2 [file mBio.01572-20-sf002.tif]

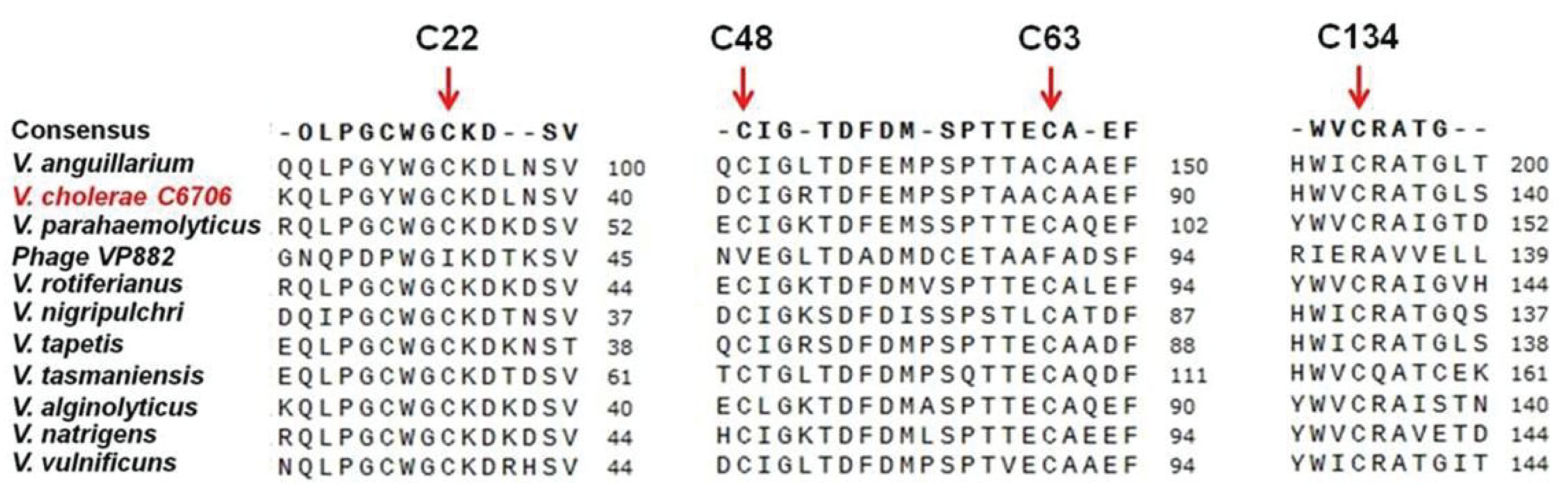

Supplement: FIG S3 [file mBio.01572-20-sf003.tif]

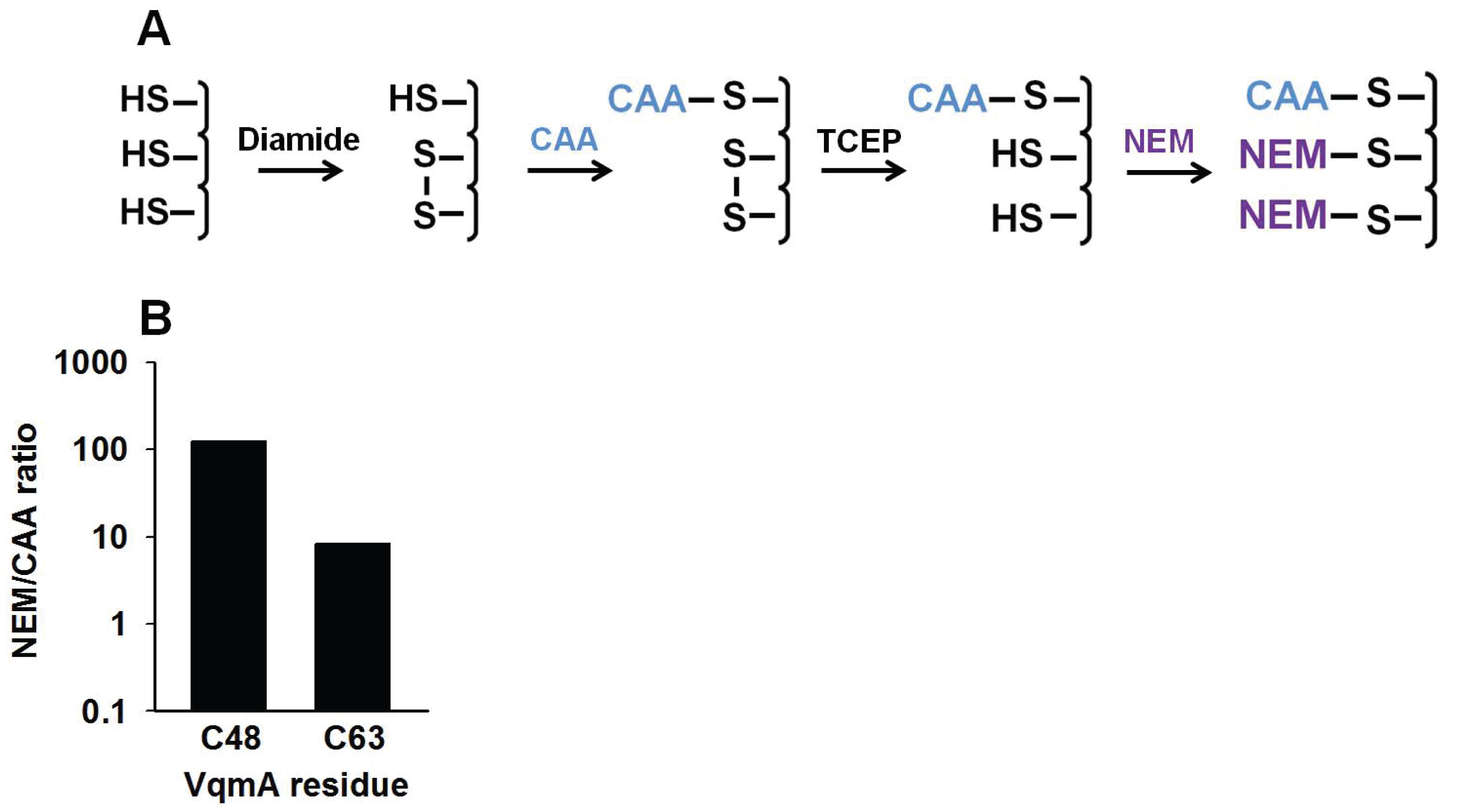

Supplement: FIG S4 [file mBio.01572-20-sf004.tif]

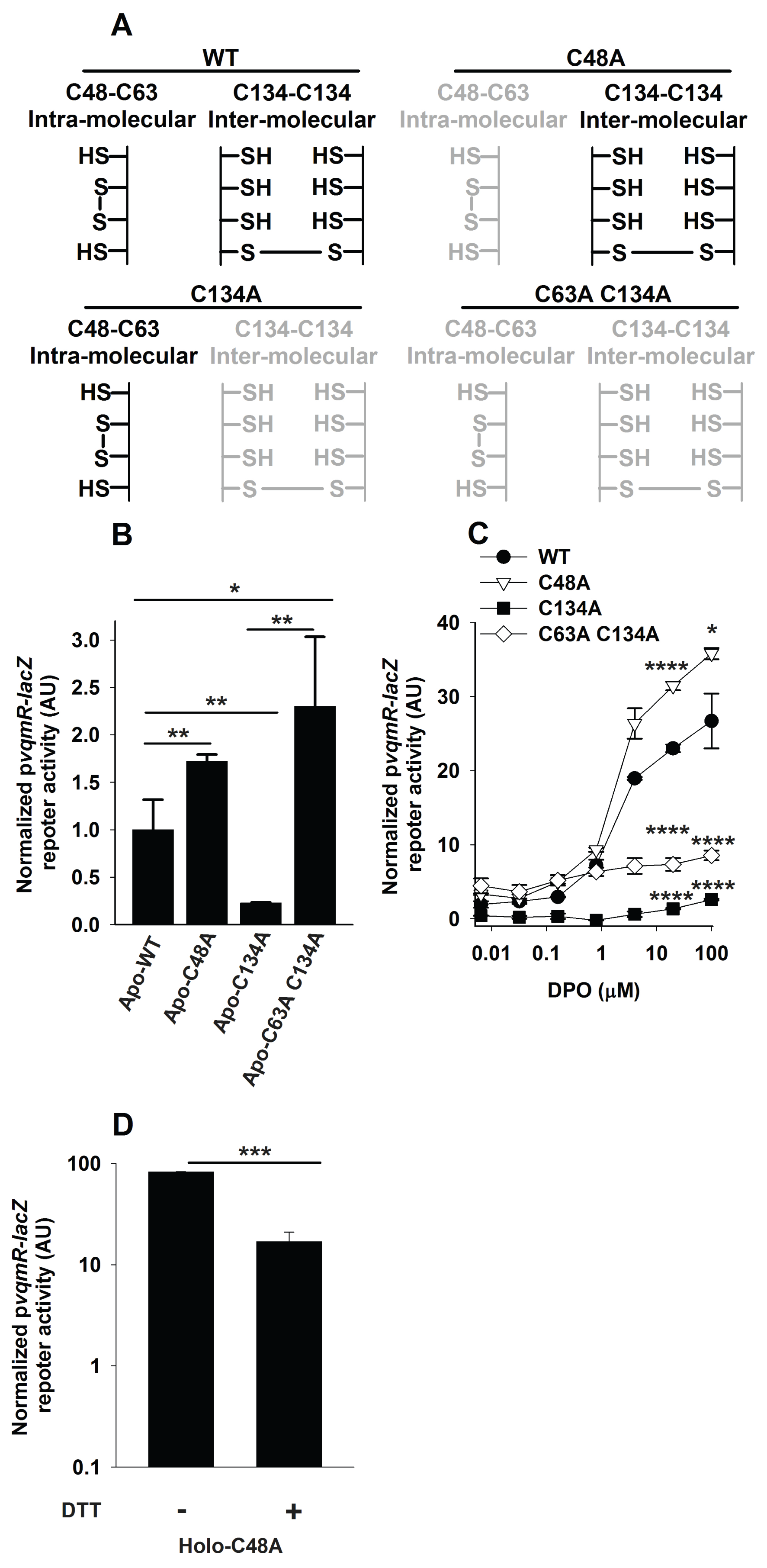

Supplement: FIG S5 [file mBio.01572-20-sf005.tif]

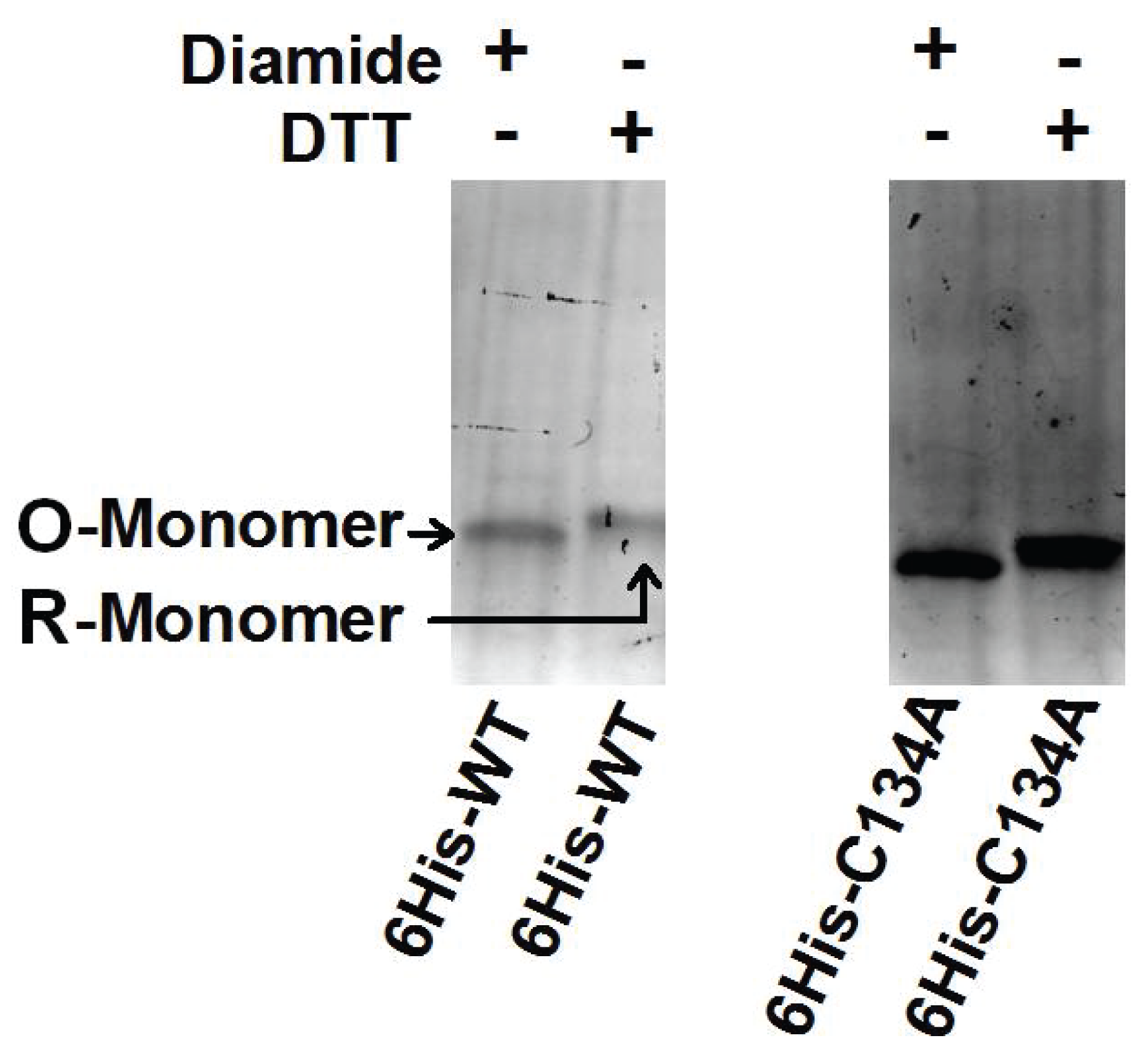

Supplement: FIG S6 [file mBio.01572-20-sf006.tif]

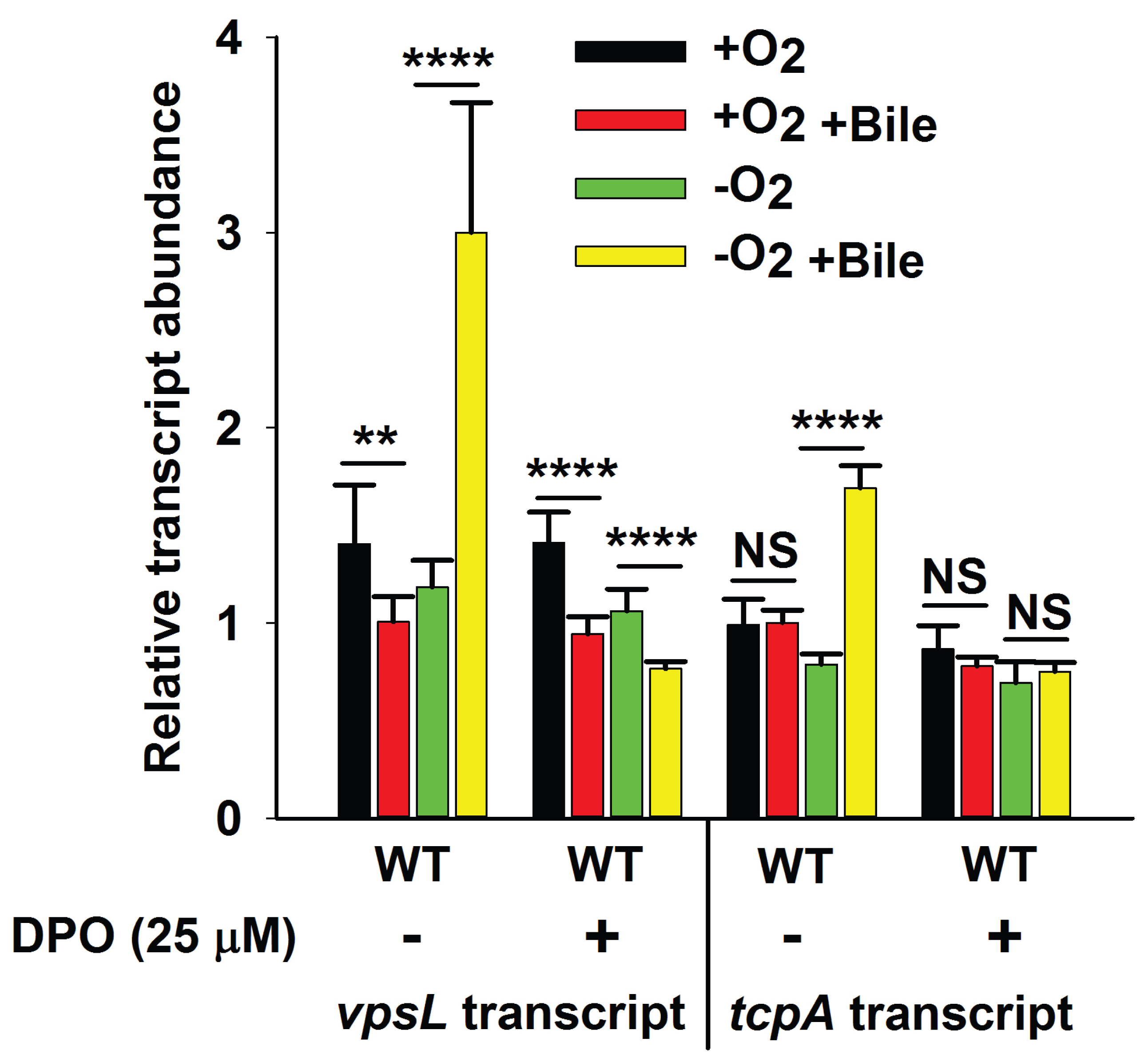

Supplement: FIG S7 [file mBio.01572-20-sf007.tif]
