# Supplementary material for: The Vibrio cholerae Quorum-Sensing Protein VqmA Integrates Cell Density, Environmental, and Host-Derived Cues into the Control of Virulence
Source: mBio. 2020 Jul 28;11(4):e01572-20. doi: 10.1128/mBio.01572-20 (PMC7387800; doi:10.1128/mBio.01572-20)
Supplement: TABLE S1 [file mBio.01572-20-st001.docx]

| **Table S1. Strains and plasmids used in this study.** | | | | | |
| --- | --- | --- | --- | --- | --- |
| **Strains used in this study** | | |  |  |  |
| **Strains** | **Genotype/Description** | | **Genetic Background** | **Source/Reference** |  |
| BB-VC 90 | *Vibrio cholerae*; Wild-type; C6706; Streptomycin resistant | | C6706 | Bassler Lab Collection |  |
| BB-VC 12 | Δ*vqmA* | | C6706 | Bassler Lab Collection |  |
| BB-VC 38 | Δ*tdh* | | C6706 | Bassler Lab Collection |  |
| BB-VC 8 | Δ*cqsA* | | C6706 | Bassler Lab Collection |  |
| BB-VC 90 | Δ*luxS* | | C6706 | Bassler Lab Collection |  |
| BB-VC 176 | Δ*cqsS* Δ*vpsS* Δ*cqsR* Δ*luxS* | | C6706 | Bassler Lab Collection |  |
| BB-VC 0325 | Δ*tdh lacZ::pvqmR-lux* | | C6706 | Bassler Lab Collection |  |
| AM 36 | Δ*cqsR* Δ*vpsS* Δ*luxQ* Δ*cqsA* | | C6706 | Bassler Lab Collection |  |
| AM3 | Δ*tdh lacZ::pvqmR-lacZ* | | C6706 | This work |  |
| AM565 | Δ*tdh vqmA::kan lacZ::pvqmR-lacZ VC1807::spec* | | C6706 | This work |  |
| AM616 | Δ*tdh* Δ*vqmA* *lacZ::pvqmR-lacZ VC1807::Cm* | | C6706 | This work |  |
| AM629 | Δ*tdh* *vqmA-flag* *lacZ::pvqmR-lacZ VC1807::spec* | | C6706 | This work |  |
| AM625 | Δ*tdh* *vqmA-C22A flag* *lacZ::pvqmR-lacZ VC1807::spec* | | C6706 | This work |  |
| AM626 | Δ*tdh* *vqmA-C48A flag* *lacZ::pvqmR-lacZ VC1807::spec* | | C6706 | This work |  |
| AM627 | Δ*tdh* *vqmA-C63A flag* *lacZ::pvqmR-lacZ VC1807::spec* | | C6706 | This work |  |
| AM628 | Δ*tdh* *vqmA-C134A flag* *lacZ::pvqmR-lacZ VC1807::spec* | | C6706 | This work |  |
| AM421 | *Escherichia coli* | | BL21 | Bassler Lab Collection |  |
| BB-EC | Δ*tdh* | | BL21 | Bassler Lab Collection |  |
| **Plasmids used in this study** | | |  |  |  |
| **Plasmid name** | | **Insert Locus/function** |  | **Source/Reference** |  |
| pEVS-pBAD | | Cloning vector for arabinose inducible expression |  | Bassler Lab Collection |  |
| pEVS-pBAD-*vqmA* *flag* | | Inducible expression & western blots |  | This work |  |
| pEVS-pBAD-*vqmA*-C22A *flag* | | Inducible expression & western blots |  | This work |  |
| pEVS-pBAD-*vqmA*-C48A *flag* | | Inducible expression & western blots |  | This work |  |
| pEVS-pBAD-*vqmA*-C63A *flag* | | Inducible expression & western blots |  | This work |  |
| pEVS-pBAD-*vqmA*-C134A *flag* | | Inducible expression & western blots |  | This work |  |
| pEVS-pBAD-*vqmA*-C63A C134A *flag* | | Inducible expression & western blots |  | This work |  |
| pET28b | | Cloning vector for protein production |  | Bassler Lab Collection |  |
| pET28b-*his6-vqmA* | | Protein production in *E. coli* |  | This work |  |
| pET28b-*his6-vqmA C134A* | | Protein production in *E. coli* |  | This work |  |
| pBB1 | | Luciferase-based quorum sensing reporter |  | Bassler Lab Collection |  |
|  | |  |  |  |  |
