# Supplementary material for: The Vibrio cholerae Quorum-Sensing Protein VqmA Integrates Cell Density, Environmental, and Host-Derived Cues into the Control of Virulence
Source: mBio. 2020 Jul 28;11(4):e01572-20. doi: 10.1128/mBio.01572-20 (PMC7387800; doi:10.1128/mBio.01572-20)
Supplement: TABLE S2 [file mBio.01572-20-st002.docx]

| **Table S2. Oligonucleotides used in this study.** | | | | |  |
| --- | --- | --- | --- | --- | --- |
| **Number** | **Oligonucleotide** | | | **Sequence** | |
| **Cloning** | | | | |  |
| AM7 | VCA1078_Mugent_For | | | ATTGAAATGCGTCTGTCGCAAATCAAACAGCG | |
| AM8 | VCA1078_Mugent_Rev | | | AAACACCGCAATCATCCCTGCGACTAG | |
| AM9 | VC1807_Mugent_For | | | TTTAAAGGGGATCAGTGACCG | |
| AM10 | VC1807_Mugent_For | | | CAATTTTGCTTTTGGACCATCCC | |
| AM41 | VCA1078_C22A_For | | | ATTACCCGGTTATTGGGGAGCCAAGGACTTAAACTCGG | |
| AM42 | VCA1078_C22A_Rev | | | CCGAGTTTAAGTCCTTGGCTCCCCAATAACCGGGTAAT | |
| AM43 | VCA1078_C48_Rev | | | AAAATCGGTGCGCCCGATGGCATCTTCAGCGCGCTTTAAG | |
| AM44 | VCA1078_C48_For | | | CTTAAAGCGCGCTGAAGATGCCATCGGGCGCACCGATTTT | |
| AM45 | VCA1078_C63_For | | | GCCTAGCCCAACAGCAGCTGCCGCTGCCGAATTTCAACAG | |
| AM46 | VCA1078_C63_Rev | | | CTGTTGAAATTCGGCAGCGGCAGCTGCTGTTGGGCTAGGC | |
| AM47 | VCA1078_C134_For | | | TGAAGTTGGTCATTGGGTCGCCCGAGCAACTGGGTTATCC | |
| AM48 | VCA1078_C134_Rev | | | GGATAACCCAGTTGCTCGGGCGACCCAATGACCAACTTCA | |
| AM269 | pBAD_up_ For | | | ACTGTTTCTCCGGATCCAAGGAGTGTATTCGTGCCTAACCATCTGACATTAGAGCAGAT | |
| AM270 | pBAD_up-rev | | | ATCTGCTCTAATGTCAGATGGTTAGGCACGAATACACTCCTTGGATCCGGAGAAACAGT | |
| AM271 | pBAD_down_for | | | TTGATTGGGCTTATGGCGCCAGTCTATCGAGGATCCGGTGATTGATTGAGCAAGCTTTA | |
| AM272 | pBAD_down_rev | | | TAAAGCTTGCTCAATCAATCACCGGATCCTCGATAGACTGGCGCCATAAGCCCAATCAA | |
|  |  | | |  | |
| **Electromobility Gel Shift Analyses** | | |  | |  |
| AM353 |  | | | TGTTGACTCAAACAATTATGCA | |
| AM360 |  | | | GGTTTGTACTTTACCGAACGCGGTA | |
|  |  | | |  | |
| **Quantitative Real-Time PCR Analyses** | | | | |  |
| AM-RT-1 | | VC1258_*gyr*_RT_For | | TGGCCAGCCAGAGATCAAG | |
| AM-RT-1 | | VC1258_ *gyr*_RT_Rev | | ACCCGCAGCGGTACGAT | |
| AM-RT-5 | | VC0934_ *vpsL*_RT_For | | CAGTATGCGAGTGATGGATAATGG | |
| AM-RT-6 | | VC0934_*vpsL*_RT_Rev | | TCGTGGATCGCCTTTGGT | |
| AM-RT-57 | | VC0828_ *tcpA*_RT_For | | GTGGTCTCAGCGGGTGTTGTTAC | |
| AM-RT-58 | | VC0828_*tcpA*_RT_Rev | | CCAAGACTACGATAAGTTTGTGTCATTGC | |
|  | |  | |  | |
