# Supplementary material for: The Vibrio cholerae Quorum-Sensing Protein VqmA Integrates Cell Density, Environmental, and Host-Derived Cues into the Control of Virulence
Source: mBio. 2020 Jul 28;11(4):e01572-20. doi: 10.1128/mBio.01572-20 (PMC7387800; doi:10.1128/mBio.01572-20)
Supplement: TEXT S1 [file mBio.01572-20-s0001.docx]

**The *Vibrio cholerae* quorum-sensing protein VqmA integrates cell density, environmental, and host-derived cues into the control of virulence**

**Authors:** Ameya A. Mashruwala^1,2^, Bonnie L. Bassler^1,2^*

**Affiliations:**

^1^Department of Molecular Biology, Princeton University, Princeton, New Jersey 08544, USA.

^2^The Howard Hughes Medical Institute, Chevy Chase, MD 20815, USA.

*Correspondence to: bbassler@princeton.edu

**Supplementary Methods**

**Supplementary Methods:**

**Analysis of CAI-1 levels following shifts in oxygen:** *V. cholerae* strains grown overnight at 37°C were diluted into fresh LB medium and incubated at 37°C with shaking to OD_600_>1 under aerobic conditions. Next, the cells were removed by centrifugation for 1 min at 13,000 rpm and a portion of the spent medium was collected. This preparation is referred to as 'Pre' in Figure S1B. The cell pellets were resuspended in equal volumes of fresh aerobic or anaerobic LB medium. A portion of the added medium was immediately removed and saved. This preparation serves as the time 0 sample in Figure S1B. The cultures were subsequently incubated in the presence or absence of oxygen, and spent media preparations were generated periodically as above. Following collection, the spent media were filtered through 0.2 μm filters. 20% (v/v) 5X LB was added to the preparations (hereafter designated as reconditioned spent medium). Subsequently, reconditioned spent medium was combined with a *V. cholerae* reporter strain that detects only exogenously supplied CAI-1 and carries a plasmid encoding the *V. harvyei luxCDABE* genes (1).

**Mass Spectrometry Data Acquisition.** Purified 6XHis-VqmA was treated with diamide followed by desalting using a C8 zip tip, dried using a SpeedVac, and dissolved in 155 µL of 50 mM ammonium bicarbonate buffer. Thereafter, accessible cysteine residues were alkylated by incubation in the presence of 18 mM CAA, at room temperature. One half of the sample was retained and subjected to mass spectrometry analysis (below). The second half of the sample was desalted, dried, and resuspended in ammonium bicarbonate buffer and cysteine residues were reduced by treatment with 10 mM TCEP and desalted. Subsequently, alkylation was achieved by incubation in the presence of 18 mM NEM. Digestion was accomplished by incubation with 500 ng of endoproteinase Glu-C overnight at 37°C.

Digested samples were dried completely in a SpeedVac and resuspended with 21 µL of 0.1% formic acid, pH 3. Thereafter, 2 µL of the samples were injected, using an Easy-nLC 1200 UPLC system, onto a 1.9 µm C18-AQ nano capillary column (Dr. Maisch, Germany; 45 cm long with 75 µm inner diameter). The columns were mated to a metal emitter in-line with an Orbitrap Fusion Lumos (Thermo Scientific, USA). Samples were resolved using a 1 h gradient (300nL/min flow rate; 45^o^C column temperature). The mass spectrometer was operated in data dependent mode with the MS1 scan conducted at a resolution of 120,000 and the following settings: positive mode, profile data type, AGC 4 x 10^5^, Max IT 54 ms, 300-1500 m/z. This procedure was followed by high energy collision dissociation (HCD) fragmentation with 35% collision energy. A dynamic exclusion list was invoked for 60 s with a maximum cycle time of 3 s to exclude previously fragmented peptides. Peptides were isolated for fragmentation using a quadrupole with a 1.2 Da window.

**Mass spectrometry data analysis.** Raw files were analyzed using the PEAKS Studio software (v. 10.0; Bioinformatics Solutions Inc) (2, 3). Parent ion and fragment tolerance were set at 70 ppm and and 0.100 Da, respectively. The oxidation of methionine residues, acetylation of protein N-termini, N-ethylmaleimide modification of cysteine residues, the conversion of glutamine residues to pyro-glutamate, and the deamidation of asparagine residues were specified as dynamic modifications. Files were searched against a database containing the His6-VqmA sequence. The VqmA sequence was obtained from the GPM database: ftp://ftp.thegpm.org/fasta/cRAP.

The Scaffold software (v. 4.8.4, Proteome Software Inc., Portland, OR) was used to validate MS/MS-based peptide and protein identifications. Peptide identification was conducted using the Scaffold Local FDR algorithm (cutoff of >90.0%). Protein probabilities were assigned using the Protein Prophet algorithm with a cutoff of >99% and a minimal requirement of least two matching peptide reads.

**References:**

1. **Miller, M. B., K. Skorupski, D. H. Lenz, R. K. Taylor, and B. L. Bassler.** 2002. Parallel quorum sensing systems converge to regulate virulence in *Vibrio cholerae*. Cell **110:**303-14.

2. **Tran, N. H., R. Qiao, L. Xin, X. Chen, C. Liu, X. Zhang, B. Shan, A. Ghodsi, and M. Li.** 2018. Deep learning enables *de novo* peptide sequencing from data-independent-acquisition mass spectrometry. Nat Methods **16:**63-66.

3. **Tran, N. H., X. Zhang, L. Xin, B. Shan, and M. Li.** 2017. De novo peptide sequencing by deep learning. Proc Natl Acad Sci U S A **114:**8247-8252.
